# Bacteria Detection at a Single-Cell Level through a Cyanotype-Based Photochemical Reaction

**DOI:** 10.1021/acs.analchem.1c03326

**Published:** 2021-12-21

**Authors:** Jiri Dietvorst, Amparo Ferrer-Vilanova, Sharath Narayana Iyengar, Aman Russom, Núria Vigués, Jordi Mas, Lluïsa Vilaplana, Maria-Pilar Marco, Gonzalo Guirado, Xavier Muñoz-Berbel

**Affiliations:** † Instituto de Microelectrónica de Barcelona (IMB-CNM, CSIC), Bellaterra (Barcelona) 08193, Spain; ‡ Nanobiotechnology for diagnostics (Nb4D), Department of Chemical and Biomolecular Nanotechnology, Institute for Advanced Chemistry of Catalonia (IQAC, CSIC), Barcelona 08034, Spain; § Departament de Química, Universitat Autònoma de Barcelona, Bellaterra (Barcelona) 08193, Spain; ∥ Division of Nanobiotechnology, Department of Protein Science, Science for life laboratory, KTH Royal Institute of Technology, Stockholm 17165, Sweden; ⊥ Departament of Genetics and Microbiology, Universitat Autònoma de Barcelona, Bellaterra (Barcelona) 08193, Spain; # CIBER de Bioingeniería, Biomateriales y Nanomedicina (CIBER-BBN), Barcelona 08034, Spain

## Abstract

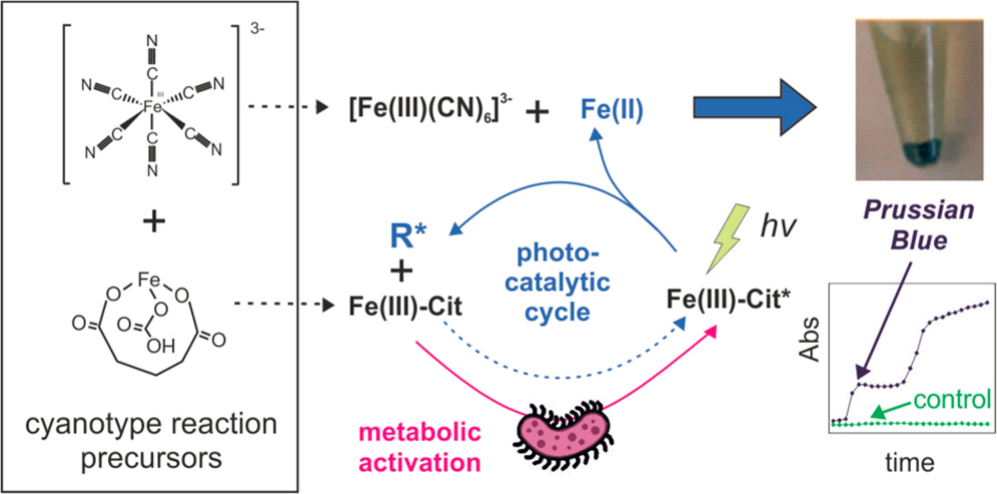

The detection of living organisms at very low concentrations is necessary for the early diagnosis of bacterial infections, but it is still challenging as there is a need for signal amplification. Cell culture, nucleic acid amplification, or nanostructure-based signal enhancement are the most common amplification methods, relying on long, tedious, complex, or expensive procedures. Here, we present a cyanotype-based photochemical amplification reaction enabling the detection of low bacterial concentrations up to a single-cell level. Photocatalysis is induced with visible light and requires bacterial metabolism of iron-based compounds to produce Prussian Blue. Bacterial activity is thus detected through the formation of an observable blue precipitate within 3 h of the reaction, which corresponds to the concentration of living organisms. The short time-to-result and simplicity of the reaction are expected to strongly impact the clinical diagnosis of infectious diseases.

## Introduction

Bacteria are microorganisms related to important healthcare and safety problems including infectious diseases,^1^ food poisoning,^2^ and water pollution.^3^ Lower respiratory infections and diarrheal diseases are now positioned as the fourth and eighth cause of death worldwide.^4^ Diarrheal diseases, resulting from the consumption of contaminated food and water, are estimated to cause 550 million foodborne illnesses and 230 000 deaths every year, where *Escherichia coli* is one of the most common foodborne pathogens.^5^ To minimize the impact of bacterial infections, these microorganisms should be detected at an early stage of infection, when only a few bacteria are present. This is particularly important in the case of sepsis, requiring the detection of less than 100 colony forming units (CFU)/mL in the bloodstream,^6^ since every hour delay in the detection of bacteria increases patients’ mortality by 10%.^7^

Current gold standard methods for microbial detection involve amplification steps to reach the appropriate bacteria detectability. The three most common amplification methods are: (i) pre-enrichment by cell culture, where bacterial proliferation increases cell population up to a detectable magnitude;^8^ (ii) nucleic acid amplification, such as the polymerase chain reaction (PCR),^9^ where a specific target sequence is copied many times in repeated cycles until reaching a detectable number of copies; and (iii) nanostructure-based signal enhancement, e.g., surface-enhanced Raman spectroscopy (SERS), where a Raman scattering signal is amplified by the use of nanoscale roughness features that increase its sensitivity up to a single bacterium level.^10^ However, the long time-to-result of pre-enrichment methods (between 24 and 36 h), the cost, and complexity of SERS and PCR^11^ limit their use in the early diagnosis of bacterial infections.

**Figure 1:**
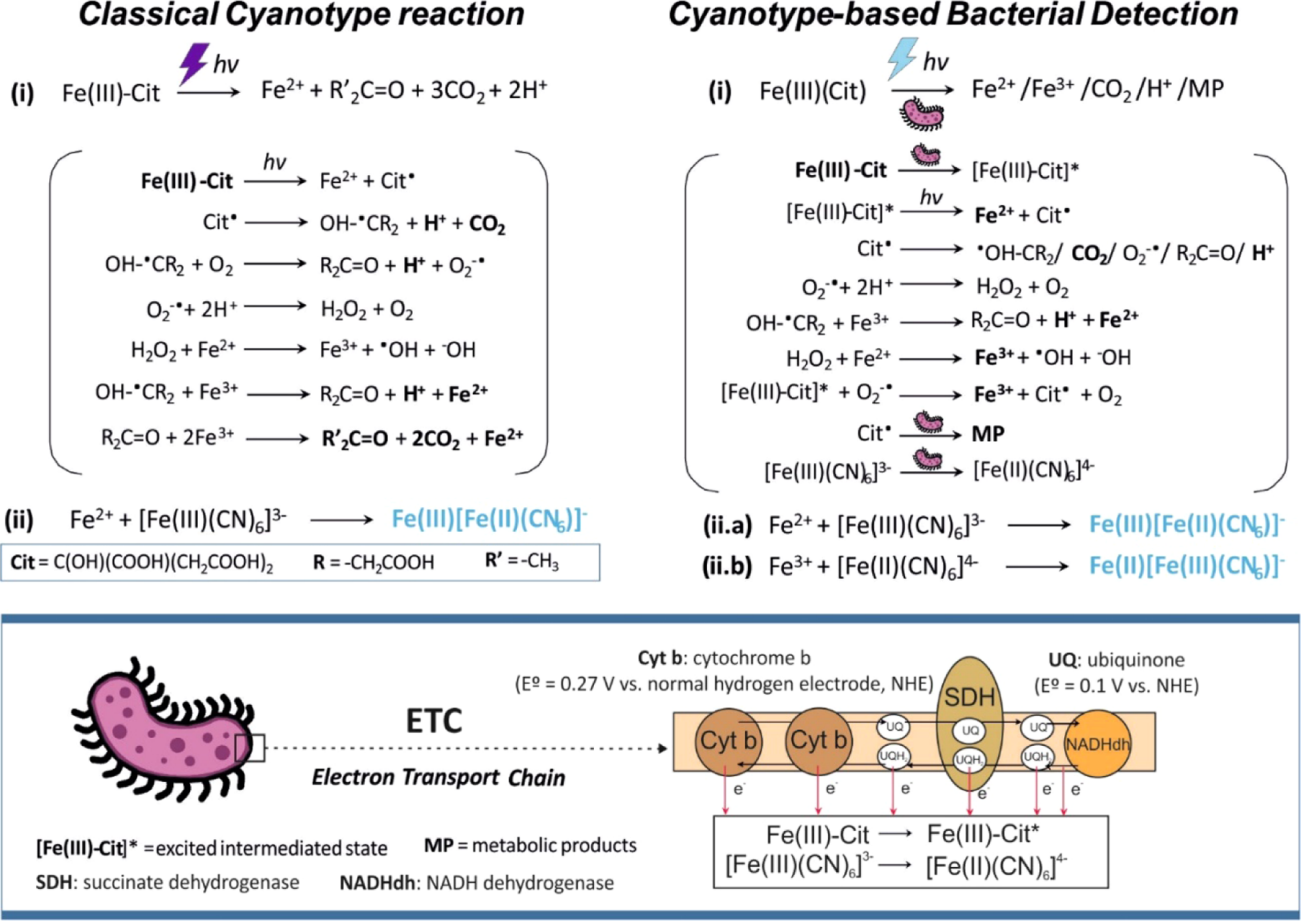
Illustration of the mechanism for the classic cyanotype (left) and the proposed cyanotype-based reaction for bacterial detection (right). In both cases, the mechanism is divided into two reactions, namely, (i) photodissociation of iron citrate and (ii) free iron ions’ reaction with hexacyanoferrate species. Below, the main components of the electron transport chain are presented with the corresponding redox potential and role in the reaction.

Conversely, photochemical reactions initiated and catalyzed by light are good candidates for bacterial detection for being fast, simple, and cheap. Among them, a cyanotype reaction is considered in the first place since employing iron-based complexes is susceptible to react with bacterial components, i.e., ferricyanide and ammonium ferric citrate. In cyanotype, UV light is used to photoactivate ammonium ferric citrate, which reacts with ferricyanide to produce Prussian Blue (PB) particles and an intense blue color.^12^ The mechanism of the reaction entails two main steps (Figure 1, left): (i) the photochemical dissociation of iron citrate complexes, involving the reduction of iron(III) to iron(II) and the concomitant oxidation of citrate;^13^ and (ii) the reaction of free iron(II) ions with ferricyanide to produce PB. It is widely accepted that citrate oxidation starts with the formation of a highly reactive citrate radical intermediate. Since poorly stable, citrate radicals dissociate rapidly to other radicals and reactive oxygen species, which react with other iron citrate molecules, accelerating their dissociation and promoting PB formation.^14^ This photocatalytic and radical mechanism makes the reaction very fast and promising for biosensing, but the need for UV radiation to initiate the reaction, which is toxic to bacteria, limits its application to bacterial detection.

In this work, a cyanotype reaction is modified in a way that UV light is substituted by less energetic visible light and coupled to bacterial metabolism for sensitive and selective detection of live bacteria.

## Materials and Methods

### Reagents

The precursor reagents for the cyanotype-based reaction were composed of ammonium ferric citrate (Sigma Aldrich) and potassium hexacyanoferrate (Sigma Aldrich). A precursor solution was prepared in Mueller Hinton (MH) media (Sigma Aldrich) and adjusted to an acidic pH of 6.5 to avoid PB decomposition.

### Bacterial Culture

*E. coli* (ATCC 25922) was donated by Prof. Herman Goossens from the University of Antwerp. Bacteria were grown overnight on an LB agar plate at 37 °C, plated using the standard streaking method. These streaks were taken from plated culture, stored at 4 °C in the fridge for a maximum of 2 weeks.

### Preparation of McFarland Standard and Bacterial Samples

The McFarland standards are employed to standardize antimicrobial susceptibility tests. The optical density of these standards is used as a reference to adjust the turbidity of bacterial suspensions used in the assay. In this case, bacterial colonies were inoculated into a sterile saline solution (0.85% NaCl w/v in water) using a cotton swab to obtain an optical density of 0.1 au at 600 nm, which corresponds to the microbiology standard McFarland standard no. 0.5. The bacterial culture was prepared to match the same optical density, 0.160 for this device, which corresponded to 10^8^ CFU/mL of bacteria. To adjust bacterial counts to the experiment of interest, the 0.5 McFarland solution was diluted in an MH medium. The optical density of the 0.5 McFarland standard was determined using a spectrophotometer (Smartspec Plus spectrophotometer, Bio-Rad, California).

### Cyanotype-Based Assays for Bacterial Detection

The assays for bacterial detection using the cyanotype reaction were based on the standard broth microdilution protocol defined by the European Committee on Antimicrobial Susceptibility Testing (EUCAST), with the small variations detailed below resulting from the photocatalytic nature of the detection reaction. The assays were performed in 96-well plates (Cell culture microplate 96 well, PS, U-bottom, Cellstar), with the optical measurements carried out using a microplate reader (Thermo-Fisher) in the range between 400 and 800 nm (step size of 10 nm) and with measurements being performed every hour. The microtiter plate was kept at 37 °C inside an incubator. Bacterial culture and reagents were transferred to the 96-well plate, for a total of 200 μL, composed of 100 μL of bacteria, 50 μL of MH, and 25 μL of each precursor to adjust the final concentration to that required in each experiment. The microtiter plate was kept at 37 °C inside an incubator, which contained a white light-emitting diode (LED) lamp (Matel 6400 K, 15 W 1500 lumen) to enable homogeneous and constant illumination to the sample from above. The sample was kept inside the illumination chamber in-between measurements. The total illumination time was 5 h, after which the plate was kept inside the heated microplate reader for the sequential automatic measurements every hour.

## Results and Discussion

**Figure 2:**
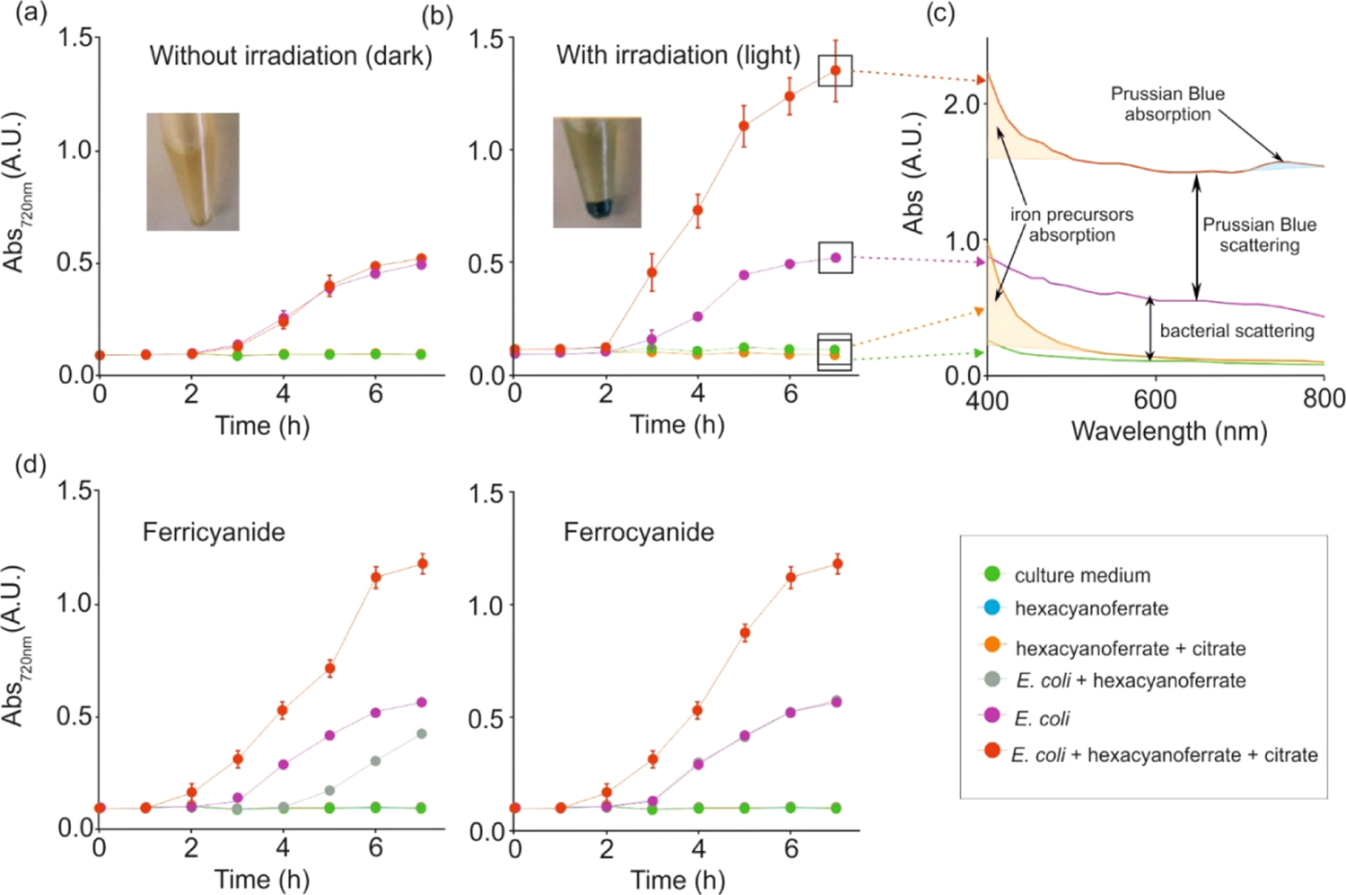
Proof of concept of the cyanotype-based reaction. Variation of the absorbance magnitude at 720 nm, corresponding to the PB peak, for samples containing the culture medium alone, cyanotype precursors alone, bacteria alone, or precursors and bacteria, either in the dark (a) or under continuous irradiation. (b) Inset, images of the aspect of the reaction Eppendorf tubes, with a clear blue precipitate in the case of light irradiation. The visible absorbance spectra of the previous light samples after 7 h of incubation are illustrated in (c), where the peaks corresponding to bacterial scattering, iron absorption, and the absorption/scattering of PB molecules are identified. In (d), the response of the cyanotype-based reaction with two hexacyanoferrate species, i.e., ferricyanide and ferrocyanide, is compared and is found to have similar absorbance values. Experimental conditions: a bacterial starting concentration of 10^6^ CFU/mL of *E. coli* ATCC 25922 was used. The reagents refer to a 1.25 mM ferricyanide and 5 mM ferric ammonium citrate concentration (*n* = 2).

Bacterial metabolism is known to reduce iron-based molecules such as ferricyanide,^15−17^ Presto Blue,^18^ or even PB^19^ by a reaction with components of the electron transport chain (ETC). Iron citrate is also selectively recognized and used by microorganisms as an iron source,^20^ where citrate has a dual role, as an iron chelator for transport of iron ions into the cell^21^ and as a key intermediate in the citric acid cycle.^22^ However, the bacterial metabolism of iron citrate alone does not release enough free iron ions to mediate the formation of PB directly. Without photoactivation of iron citrate (i.e., dark conditions shown in Figure 2a), the spectroscopic analysis of PB at 720 nm (corresponding to its absorption peak) does not show PB formation either in the presence or the absence of bacteria. The increase in absorbance reported in the dark samples containing bacteria is associated with cell scattering and follows a typical proliferation curve for *E. coli*. Since both bacterial samples with and without cyanotype precursors show similar growth curves, it is evident that these reagents do not compromise bacterial growth at the experimental conditions under study.

In light conditions (Figure 2b), ultraviolet (UV) radiation used in classical cyanotype is substituted by less energetic visible light, which is not sufficient to directly catalyze the photochemical dissociation of iron citrate necessary for the production of PB (orange curve in the figure). PB formation is only evident when both cyanotype precursors and bacteria are present and continuously irradiated. PB appears as a blue precipitate (image inset the figure) and results in a large increase in the absorbance magnitude at 720 nm, which is associated with the sum of the scattering and absorption of PB particles (Figure 2c). When analyzing the contribution of each reaction component to the formation of PB, it is evident that both iron citrate and hexacyanoferrate molecules are necessary (Supporting Information, S1). However, the reaction mechanism does not depend on the oxidative state of the hexacyanoferrate ions and similar results are obtained when substituting ferricyanide by its reduced form ferrocyanide (Figure 2d). Considering that the formation of PB requires the presence of free iron ions with a specific oxidative state to react with hexacyanoferrate complexes, i.e., iron(II) in the case of ferricyanide and iron(III) for ferrocyanide, this result suggests that both iron(II) and iron(III) ions are produced after photometabolic dissociation of iron citrate molecules. However, at this stage, it has not been possible to confirm the presence of free iron(III) (Supporting Information, S2). Iron(III) detection is based on the specific reaction between acetylsalicylic acid and free iron(III) ions, resulting in the formation of a complex with an intense purple color and absorbance at 565 nm. Complex formation has not been observed in any of the previous reaction conditions. The reason for this may be a fast reaction kinetic between free iron(III) and hexacyanoferrate ions to form PB and thus, a short lifetime of free iron ions. In addition, the lack of a reaction between ferrocyanide and iron citrate in light conditions (Figure 2d, orange line in the ferrocyanide plot), which should produce PB directly through the classical cyanotype reaction, confirms the slow kinetics of the photochemical dissociation of iron citrate molecules with visible light.

Considering previous results, the reaction mechanism presented in Figure 1 right is proposed. It involves the diffusion of iron citrate into the periplasmic region of bacteria, where it is excited to an intermediate state [Fe(III)-Cit]° by proteins and mediators of the bacterial ETC, e.g., cytochromes and ubiquinone. Thus, the intermediate formation requires intact bacterial membranes, so the presence of viable bacteria starts the reaction. This excited intermediate is susceptible to photochemical dissociation by visible light, releasing iron(II) ions and other radical intermediates, resulting from the photo-oxidation of citrate to the medium. Once dissociated, a photocatalytic cascade begins, where radicals react with other iron citrate molecules inducing their dissociation. Part of the iron(II) ions are reoxidized to iron(III) by the effect of the radicals and hydrogen peroxide produced as side products by the reaction. Bacterial metabolism additionally reduces intermediate iron compounds, metabolizes citrate, and reduces ferricyanide to ferrocyanide. Therefore, independently of the initial composition of the cyanotype precursor solution, the photometabolic activation of the reagents results in a mixture of free iron(II) and iron(III) ions, ferricyanide and ferrocyanide, resulting in the fast formation of PB molecules.

**Figure 3:**
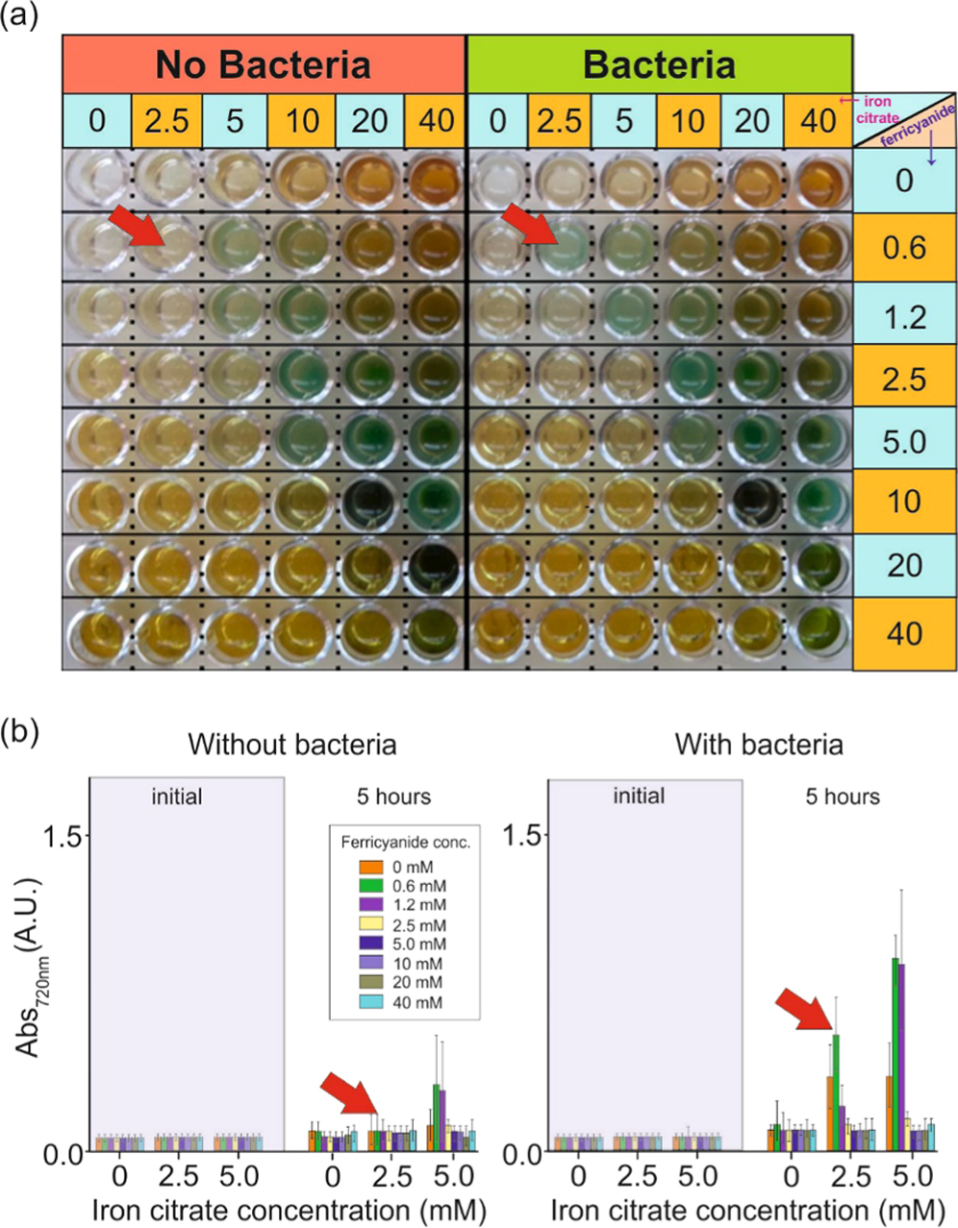
Optimization of the composition of the cyanotype precursor solution. (a) Image illustrating the color of the reaction solution after 5 h of incubation with several precursor solutions dilutions and reagents proportions. The absorbance magnitude of the most representative samples is illustrated in (b). Red arrows indicate the difference between the control sample without bacteria and the sample containing 5 × 10^5^ CFU/mL *E. coli* ATCC 25922 for the concentration and proportions considered optimal (*n* = 3).

This photometabolic activation only takes place when using diluted cyanotype precursor solutions containing iron citrate concentrations below 10 mM and maintaining a molar ratio citrate:hexacyanoferrate in the range between 4 and 8 (Figure 3a; above 10 mM iron citrate, a classical cyanotype reaction occurs, where PB is formed spontaneously without the need for bacteria). From all combinations under study, the proportion 2.5:0.62 mM iron citrate:hexacyanoferrate presents the highest signal-to-noise ratio for being the one with the lowest background noise, which corresponds to the signal of the control samples without bacteria (Figure 3b; the absorbance magnitude and spectra of all conditions are presented in the Supporting Information, S3).

**Figure 4:**
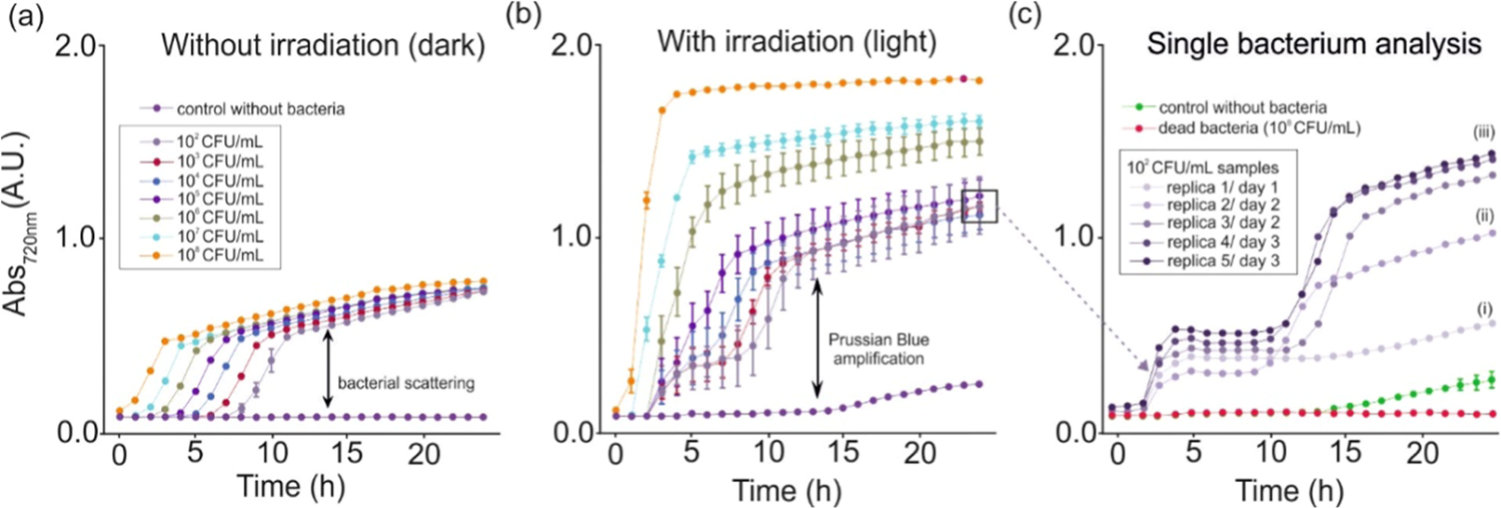
Evaluation of the response of the cyanotype-based reaction with different bacterial concentrations. Variation of the absorbance magnitude at 720 nm for samples containing cyanotype precursor solutions and bacteria at concentrations between 100 and 10^8^ CFU/mL in the dark (a) or after 5 h of continuous irradiation (b) (*n* = 3). In (c), the replicas of the 100 CFU/mL sample are represented individually to evaluate the variability in the single bacterium detection and compared to controls without bacteria and with dead bacteria.

When the photometabolic reaction is conducted at different bacterial concentrations (Figure 4b), high signal amplification is obtained in all samples. A particular behavior is observed in samples below 10^5^ CFU/mL, where bacterial activity is slower than photocatalysis. In this case, a sudden increase in absorbance resulting from PB formation is reported within 3 h of the reaction, followed by stabilization and a second increase attributed to bacterial scattering.

This fast PB formation is independent of the bacterial concentration for samples below 10^5^ CFU/mL and selective since it only happens in light samples containing bacteria and not in the dark ones (Figure 4a) or controls without bacteria. Therefore, the photocatalytic reaction is much faster than bacterial metabolism, which is in agreement with the mechanism proposed in Figure 1 right. That is, bacteria initiate the reaction but, after that, the light becomes responsible to produce Cit°, other radicals, and reactive oxygen species in a cascade reaction, resulting in the production of free iron ions and PB molecules. This reaction takes place in 3 h in samples’ containing bacterial concentrations as low as 100 CFU/mL, which correspond to an average of 10 CFUs for 100 μL of samples. The assay, however, reaches single bacterium sensitivity and is highly repetitive, as shown in Figure 4c. In the figure, samples are grouped according to the initial bacterial concentration in (i) low bacteria (between 1 and 2 CFUs), (ii) middle (between 5 and 8 CFUs), and (iii) high (above 10 CFUs). It is clear from the results that one to two bacterial cells are not enough to induce bacterial proliferation since the second absorbance increase associated with bacterial scattering is absent in this case. Control samples without bacteria or containing dead bacteria do not present the initial sudden increase, confirming that this is a photometabolic process only happening when live bacteria are present at the initial stages of the reaction (even if they die afterward). However, the control without bacteria is not completely flat but presents some increase that may be due to some late reaction between precursor components (after 11 h) to produce PB (e.g., direct cyanotype reaction). The cyanotype-based reaction has no cross-reactivity with components present in the serum, and blood (Supporting Information, S4) since there is no PB formation in those serum/blood samples that do not contain bacteria. However, the reaction in these complex matrices requires the optimization of the composition of the precursor solution to be able to detect PB formation within 3 h (now requiring 5 h for 10^2^ CFU/mL samples). It is important to remark that the photochemical reaction has been demonstrated for *E. coli*, which is used as a model microorganism. However, proteins and redox mediators present in the ETC are very conservative between bacterial species, and such a reaction should be applicable to a wide number of Gram-positive and Gram-negative bacteria. In this sense, the reaction has been already demonstrated by the Gram-positive microorganism *Staphylococcus aureus* in a recent article of the group focused on sepsis diagnostics.^23^

## Conclusions

In summary, the substitution of UV by visible light in a cyanotype photochemical reaction enables the coupling of PB formation to bacterial metabolism. The reaction mechanism involves the photoactivation of iron citrate molecules into an excited intermediate state by reactive components in the electron transport chain, which is susceptible to photoactivation with visible light to produce free iron ions and citrate radicals. Iron ions react with hexacyanoferrate molecules to produce PB particles in less than 3 h, a process catalyzed by citrate radicals that initiate a cascade reaction with other iron citrate molecules to produce more radicals and free iron ions. The reaction is very selective, requiring the presence of light and bacteria, and sensitive since it may be activated by a single microorganism. Components of complex biological fluids such as serum or blood do not present cross-reactivity with the reaction precursors, making it possible to detect very low bacterial concentrations in a short time and without sample pretreatment. The simplicity, selectivity, and short time-to-result of this photometabolic reaction are envisioned to have an impact on clinical diagnosis of bacterial infections, in particular in sepsis diagnostics where early detection of bacteria in the bloodstream is fundamental to improve the prognosis of the pathology. This protocol may be also used for fast antibiotic susceptibility testing, the determination of minimal inhibitory concentration, and the identification of resistant bacteria.

## Supporting Information Available

Additional experiments for the evaluation of the role of cyanotype precursors in the reaction (S1); additional experiments for the evaluation of the presence of free iron(III) molecules during the cyanotype-based reaction (S2); additional experiments to study the proportion and concentration of the precursor solution components (S3); additional experiments to evaluate the cross-reactivity of the cyanotype-based reagent with serum and blood components (S4) (PDF)
