# Supplementary material for: Bacteria Detection at a Single-Cell Level through a Cyanotype-Based Photochemical Reaction
Source: Anal Chem. 2021 Dec 21;94(2):787–92. doi: 10.1021/acs.analchem.1c03326 (PMC8771638; doi:10.1021/acs.analchem.1c03326)
Supplement: Supplementary file 1 — ac1c03326_si_001.pdf [file ac1c03326_si_001.pdf]

Supplementary Information

Bacteria detection at single-cell level through a cyanotype-based photochemical reaction

Jiri Dietvorst,[a],[b] Amparo Ferrer-Vilanova,[a],[c] Sharath Narayana Iyengar,[d] Aman Russom,[d] Núria Vigués,[e] Jordi Mas,[e] Lluïsa Vilaplana,[b] Maria Pilar Marco,[b] Gonzalo Guirado*[c] and Xavier Muñoz-Berbel,*[a]

[a] Jiri Dietvorst, Amparo Ferrer-Vilanova, Dr. Xavier Muñoz-Berbel
Instituto de Microelectrónica de Barcelona (IMB-CNM, CSIC), Bellaterra (Barcelona), 08193, Spain
E-mail: xavier.munoz@imb-cnm.csic.es

[b] Jiri Dietvorst, Dr. Lluïsa Vilaplana, Prof. Maria Pilar Marco
Nanobiotechnology for diagnostics (Nb4D), Department of Chemical and Biomolecular Nanotechnology, Institute for Advanced Chemistry of Catalonia (IQAC, CSIC), Spain
Networking Research Center of Bioengineering, Biomaterials and Nanomedicina (CIBER-BBN)

[c] Amparo Ferrer-Vilanova and Dr. Gonzalo Guirado
Departament de Química, Universitat Autònoma de Barcelona, Bellaterra (Barcelona), 08193, Spain
E-mail: Gonzalo.Guirado@uab.cat

[d] Sharath Narayana Iyengar and Prof. Aman Russom
Division of Nanobiotechnology, Department of Protein Science, Science for life laboratory, KTH Royal Institute of Technology, Stockholm, Sweden, 17165

[e] Dr. Núria Vigués and Prof. Jordi Mas
Departament of Genetics and Microbiology, Universitat Autònoma de Barcelona, Bellaterra (Barcelona), 08193, Spain

<u>Supplementary information S1:</u> Evaluation of the role of cyanotype precursors.

Due to the importance of understanding each and any interaction between the various reagents of the photochemical reaction, experiments were conducted simultaneously in light and dark and using controls for every combination of reagent, which used at the optimal concentration determined previously. The tests were run for a total of 5 hours, and measurements were taken every hour.

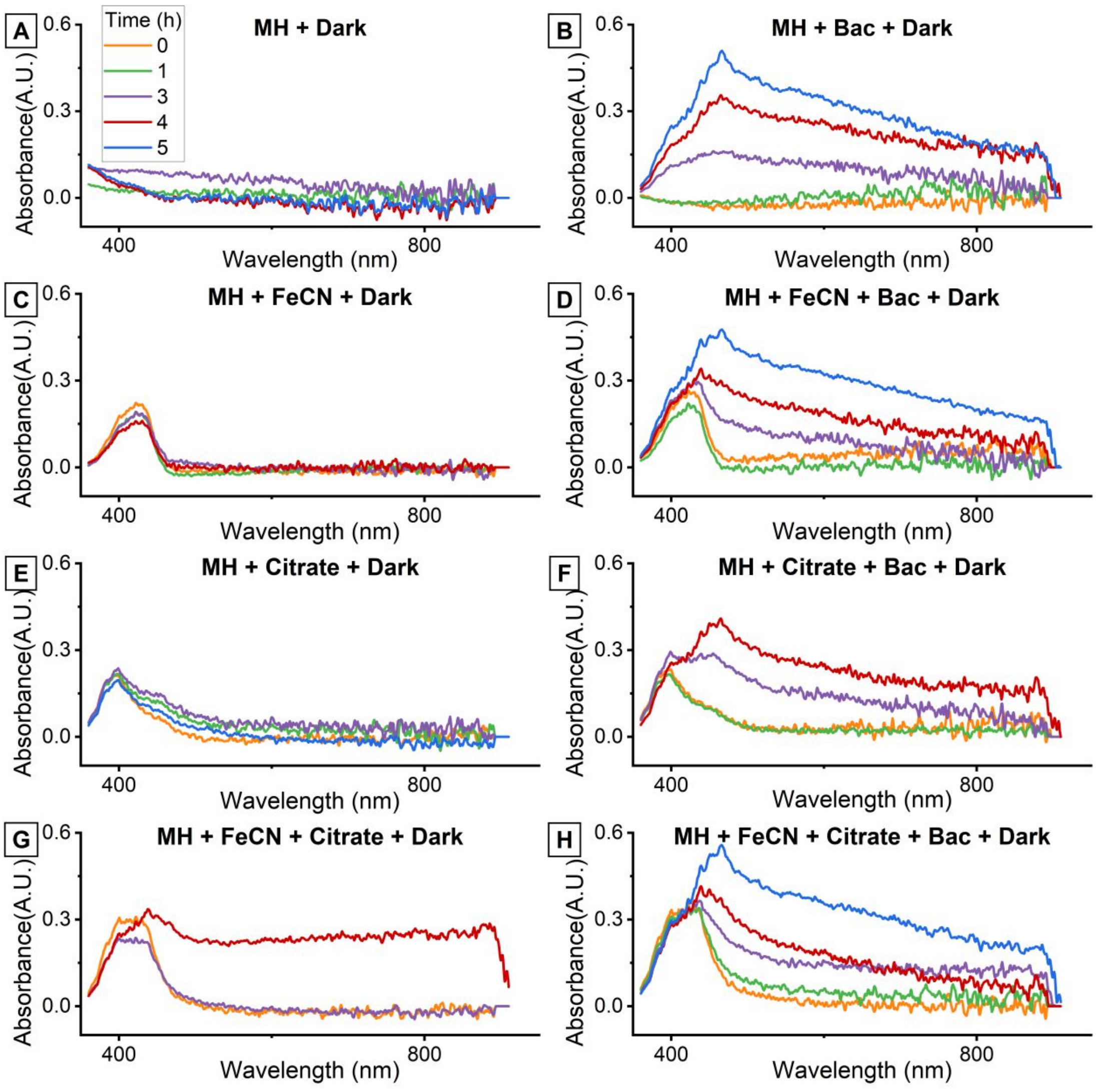

Fig. S1. Evaluation of performance and reaction between the various reagents of the cyanotype-based protocol performed in dark. *Measurements were taken every hour, for a total of 5 hours, at every nm between 350 nm and 900 nm. For the cyanotyping, a 2.5 mM iron (III) citrate and a 0.625 mM ferricyanide were used. A bacterial concentration of 5x10^5 CFU/mL of E. coli ATCC 25922 was used. Lines missing in the plots were omitted due to instrumental errors. (n=1)*

In the figures, MH corresponds to Mueller-Hinton culture medium, Bac to bacteria, FeCN to ferricyanide and citrate to iron citrate. The first set of experiments was performed without illumination to evaluate cross-reactivity between reagents, as summarized in **Fig. S1**. The four graphs on the left show the lack of reactivity between MH culture medium, ferricyanide and iron citrate since these plots did not present changes in the absorbance spectra over time. Only minor changes were observed in **graph G** after 4 hours of incubation, which were attributed to some contamination. The four graphs on the right replicated the experiments in the left but including bacteria to study their influence in the reaction. The same spectral variation was observed by the four plots, which was due to bacterial scattering. The impact of biomass scattering on absorbance spectra was very important (i.e. broadband change in the full wavelength range under study) and prevented the observation of additional changes associated to other reactions between reagents. However, due to the similarity between **plot B**, only containing bacteria proliferating in culture medium, and the other three plots, it may be concluded that it was not Prussian blue formation in any of the experimental conditions previously studied, demonstrating that the light was key for the activation of the photochemical reaction.

The same experiment was performed simultaneously, but with continuous visible light illumination, with the results summarized in **Fig. S2**. Interestingly, no Prussian Blue formation was observed in the four plots in the left, where all reagents were combined and continuously irradiated in absence of bacteria. Thus, although the mechanism was intrinsically photocatalytic, the presence of light was not enough to activate the photochemical reaction and Prussian Blue formation. The most remarkable observation in the plots in the left (without bacteria) was that the absorbance peak attributed to ferricyanide (420 nm) slightly decreased over time, probably due to photochemical degradation (**graph C and G**). When bacteria are present (plots in the right), the spectral response depended on the composition of the medium. Thus, the same increase associated to a conventional bacterial proliferation obtained in the experiments performed in the dark is replicated during irradiation by **graphs B**, only containing bacteria and MH culture medium, and **F**, incorporating iron citrate. This indicated that light, bacteria and iron citrate was not enough to

activate the photocatalytic formation of PB. Similarly, the **graph D**, resulting from the reaction of ferricyanide and bacteria in light conditions, presented a bacterial proliferation even lower than in the dark. This confirmed the previous hypothesis suggesting that light may induce some photo-degradation of ferricyanide, probably inducing the release of cyanide ions that killed bacteria and reduce their proliferation. This was in agreement with previous publication by Ojeda et al. (https://doi.org/10.1039/C7CP03337K) that reported on the liberation of cyanide ions from the ferricyanide molecule under UV-VIS illumination. Finally, the combination of iron citrate, ferricyanide and bacteria under continuous irradiation catalysed Prussian blue formation, as shown in **graph H**.

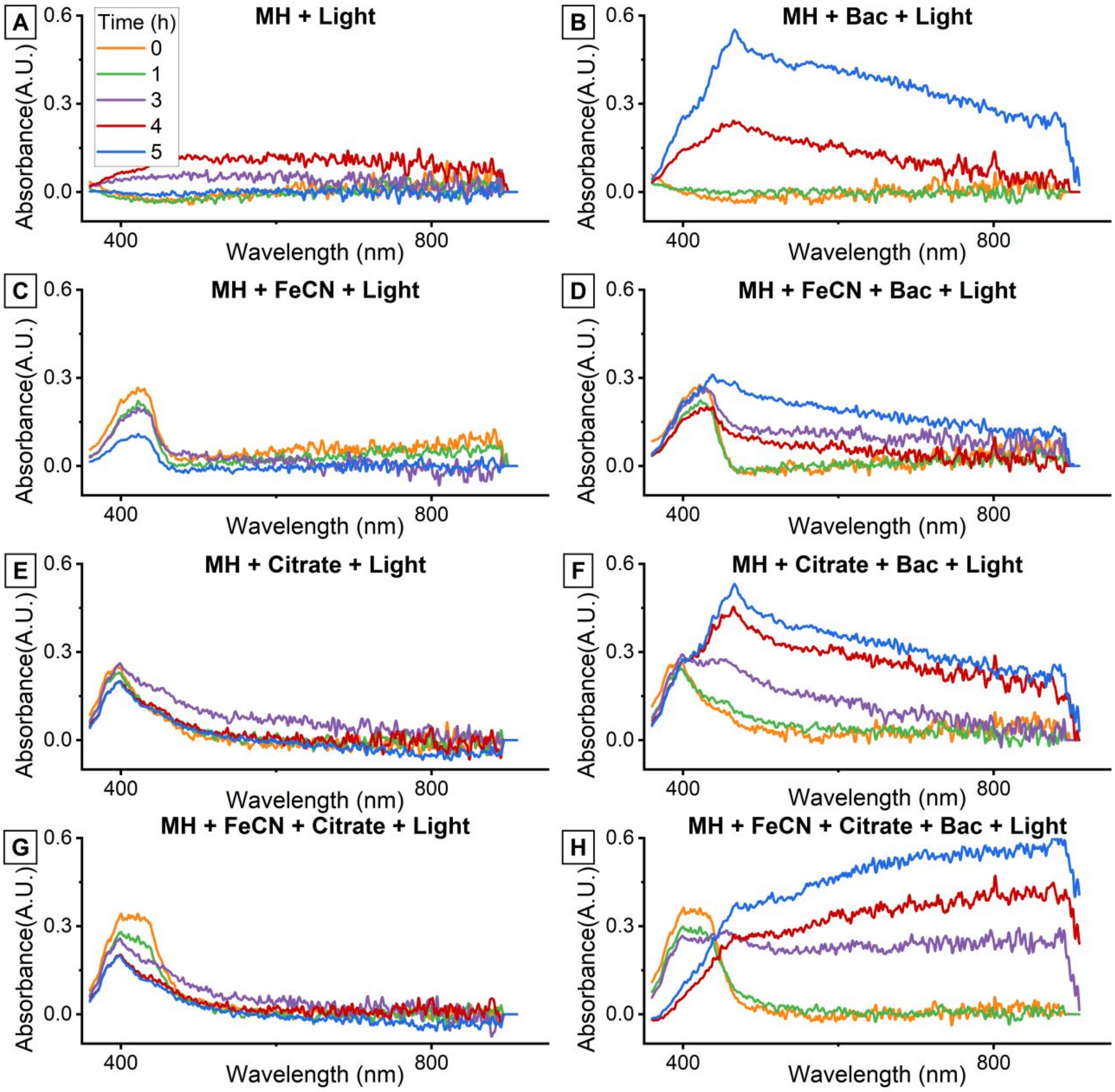

Fig. S2. Evaluation of performance and reaction between the various reagents of the cyanotype-based protocol performed under continuous illumination. *Measurements were taken every hour, for a total of 5 hours, at every nm between 350 nm and 900 nm. For the cyanotyping, a 2.5 mM iron (III) citrate and a 0.625 mM ferricyanide were used. A bacterial concentration of $5x10^5$ CFU/mL of E. coli ATCC 25922 was used. Lines missing in the plots were omitted due to instrumental errors. (n=1)*

Supplementary information S2: Evaluation of the presence of free iron (III) molecules during the cyanotype-based reaction.

The presence of free iron ions was of key relevance to elucidate the mechanisms of the cyanotype-based photochemical reaction here described, but not possible to attain through spectroscopy directly. For this reason, an additional experiment was performed to evaluate the oxidative state of the iron species produced in the photochemical reaction from the ferric ammonium citrate used as free iron source. The experiment performed is summarized in **Fig. S3**. In the experiment, iron nitrate ($Fe(NO_3)_3$) was used as source of free iron (III) ions in the positive controls. The reaction mechanism considered the formation of a complex between the free iron(III) ions and acetylsalicylic acid (AA), which presented an intense purple colour and a clear absorbance band at 565 nm (**Fig. S3A**).

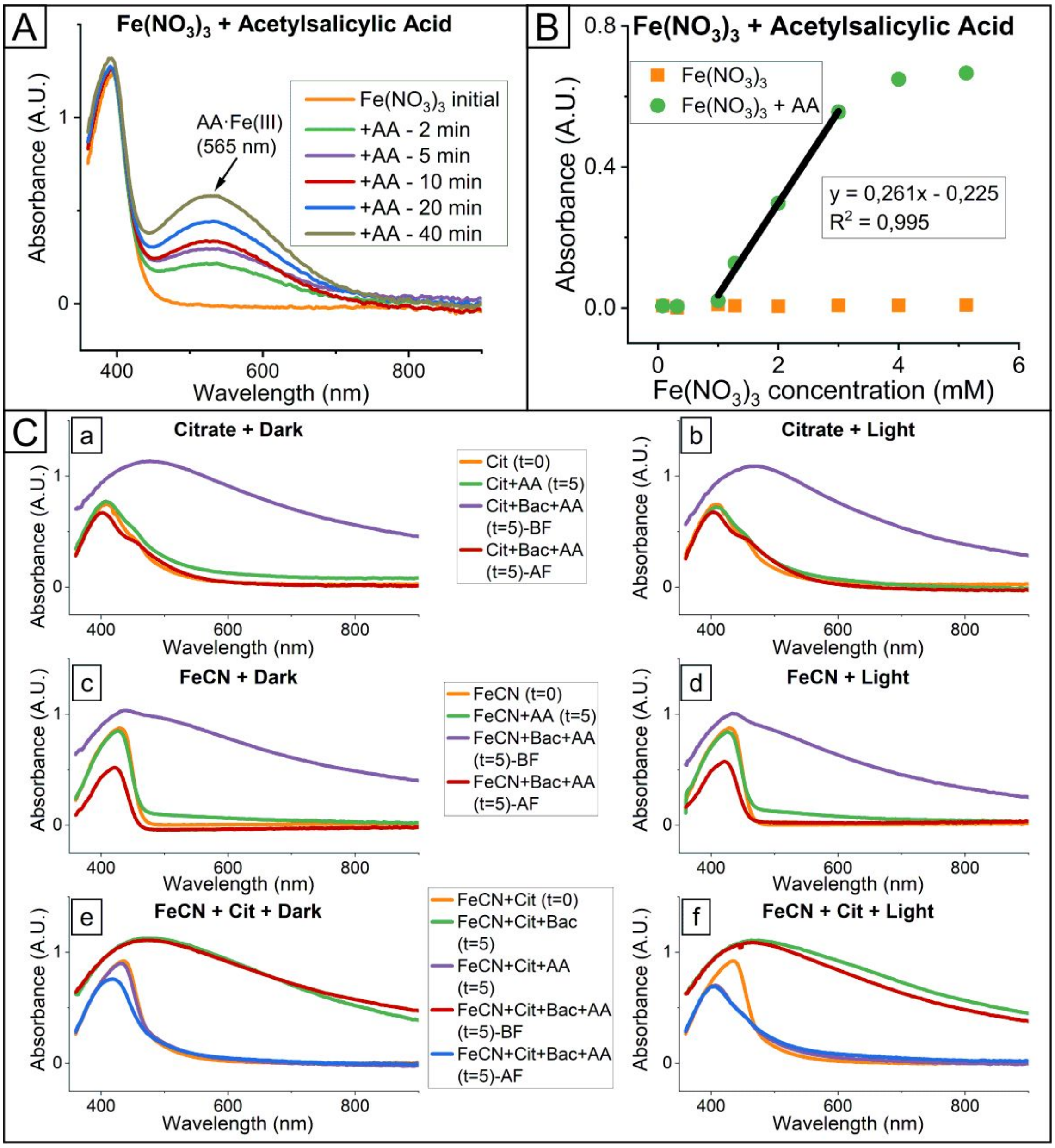

Fig. S3. Acetylsalicylic acid experiments for the detection of free iron (III). *A) acetylsalicylic acid (AS) test using iron nitrate as reference. Measurements were performed after the indicated reaction time with AS and taken every nm from 350 – 900 nm. B) Calibration curve of the spectral response of free iron from iron nitrate after reacting with AS for 40 min, measured at 565 nm. C) Detection of free iron from the reagents of the cyanotype-based reaction, measured after 5 hours of incubation in dark or after continuous illumination. Measurements taken every nm from 350 – 900 nm. FeCN = Ferricyanide, Cit = ferric ammonium citrate, Bac = Bacteria, AA = acetylsalicylic acid, BF = measurement before filtration of the sample, AF = measurement after filtration of the sample (filter = 200 nm pore size).*

Graph A highlights the increase in absorbance over time of the reaction. Based on the experiment, 40 minutes of reaction was taken as optimal for the reaction. The magnitude of the absorbance band was proportional to the free iron (III)

concentration in a range between 1 and 3 mM iron (III). The calibration curve is shown in **graph B**, and results in a LOD of 0.269 mM. Considering the final concentrations of ferricyanide and iron citrate in the cyanotype photochemical reaction (0.625 and 2.5 mM, respectively) the sensitivity of the method should be enough to detect the presence of free iron (III) ions.

Based on the latter, the AA was added to the cyanotype reaction to check for the presence of free iron (III) ions during the reaction. To account for the influence of bacteria on the AA reaction, the samples were also measured before (BF) and after filtering (AF) of the bacteria from the sample. The results of the AA test with various reagents with and with bacteria and with and without light are summarized in **Fig. S3 C**. None of the reactions studied presented a peak at 565 nm indicative of the presence of free iron (III) ions in the medium at concentrations above 0.3 mM. The drop in absorbance around 420 nm using ferricyanide and bacteria after 5 hours was due to bacteria being able to reduce ferricyanide. Additionally, if no filtration was performed, the spectra were dominated by the bacterial scattering.

Due to impossibility to measure free iron (III) ions, two hypotheses were suggested: free iron (III) were released and rapidly reacted with iron-cyanide complexed to form Prussian Blue.

<u>Supplementary information S3:</u> Study of the proportion and concentration of the precursor solution components.

In cyanotype, ferric ammonium citrate concentrations ranging from 0 to 40 mM were tested against ferricyanide solutions containing between 0 and 40 mM, both in dark and in light. Data belonging to time 0 and after 5 hours of incubation in $5x10^5$ CFU/mL of *E. coli* ATCC 25922 are shown in **Fig. S4** and **Fig. S5,** corresponding to experiments in the dark and light, respectively. The results were measured every hour, for a total of 24 hours, over the entire visible spectrum, with the absorbance plotted at 720 nm. Only four conditions for each experiment are plotted in the figures for clarity.

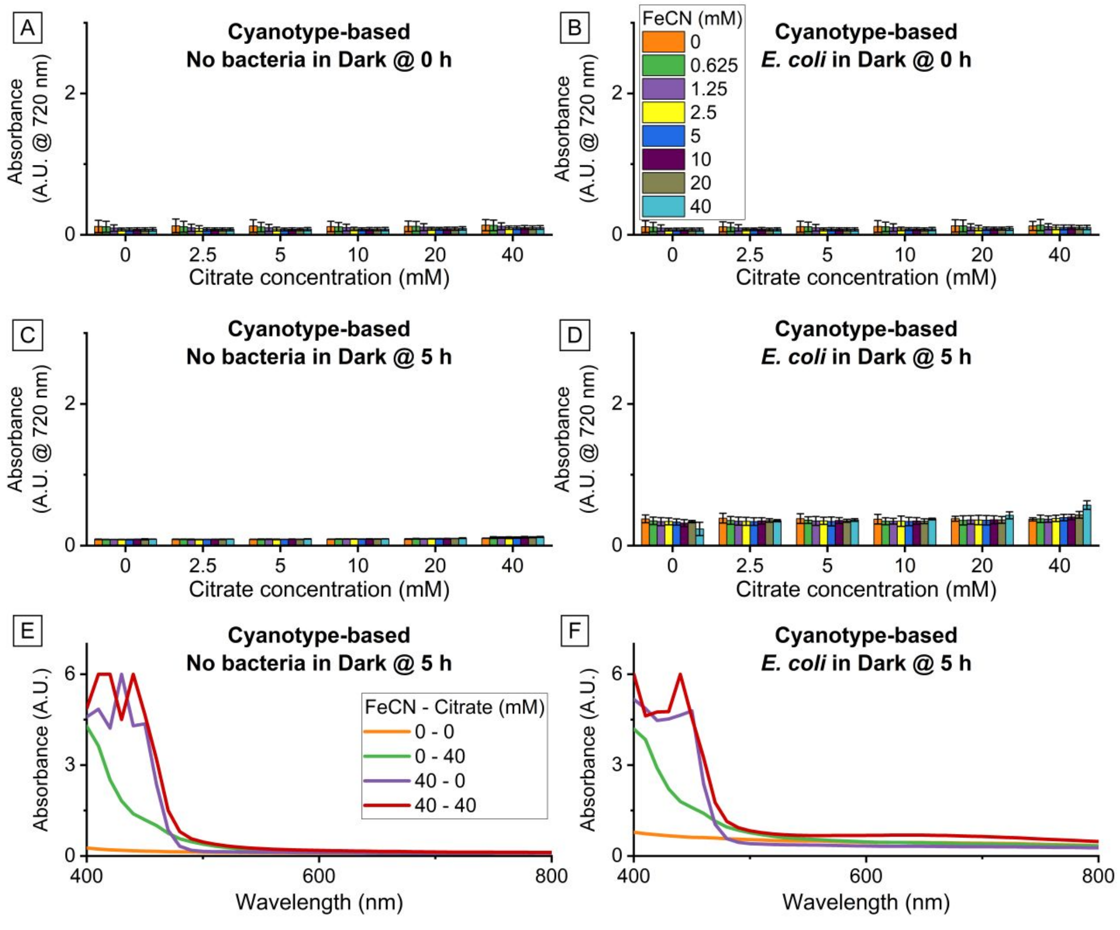

Fig. S4. Cyanotyping optimization under dark conditions. *A) No bacteria are present, with an incubation time of 0 hours. B) $5x10^5$ CFU/mL of* E. coli *ATCC 25922 with an incubation time of 0 hours. C) No bacteria are present, with an incubation time of 5 hours. D) $5x10^5$ CFU/mL of* E. coli *ATCC 25922 with an incubation time of 5 hours. E) Absorbance spectra for 4 conditions, without bacteria, after an incubation time of 5 hours. F) Absorbance spectra for 4 conditions, with $5x10^5$ CFU/mL of* E. coli *ATCC 25922, after an incubation time of 5 hours Each group of*

columns refers to a single ferric ammonium citrate concentration. Each colour corresponds to a specific ferricyanide concentration. Data is plotted at a wavelength of 720 nm for A – D. (n=3)

In the absence of light, the lack of PB formation at any of the conditions was apparent. The constant signal for any of the reagent concentrations in the absence of bacteria indicated that the reagents did not react with either the media or between each other. When bacteria were present, no apparent PB formation was observed either. The only exception was when combining 40 mM citrate with a 40 mM FeCN concentration. The high concentration of both reagents in the precursor solution led to the formation of PB, even if it was very little (see the small band at 720 nm shown in **Fig. S4 F**).

The absorbance in samples with bacteria was higher than without (**Fig. S4 E and F**), which was associated to bacterial scattering. Although there were no significant differences between absorbance values at any of the reagent conditions ($p<0.05$), in the case of the precursor solution containing 40 mM FeCN without citrate, a slight reduction in absorbance was observed. This may be attributed to some toxicity of the ferricyanide when used at high concentrations, as is the case here, which limited bacterial proliferation, reducing the magnitude of bacterial scattering.

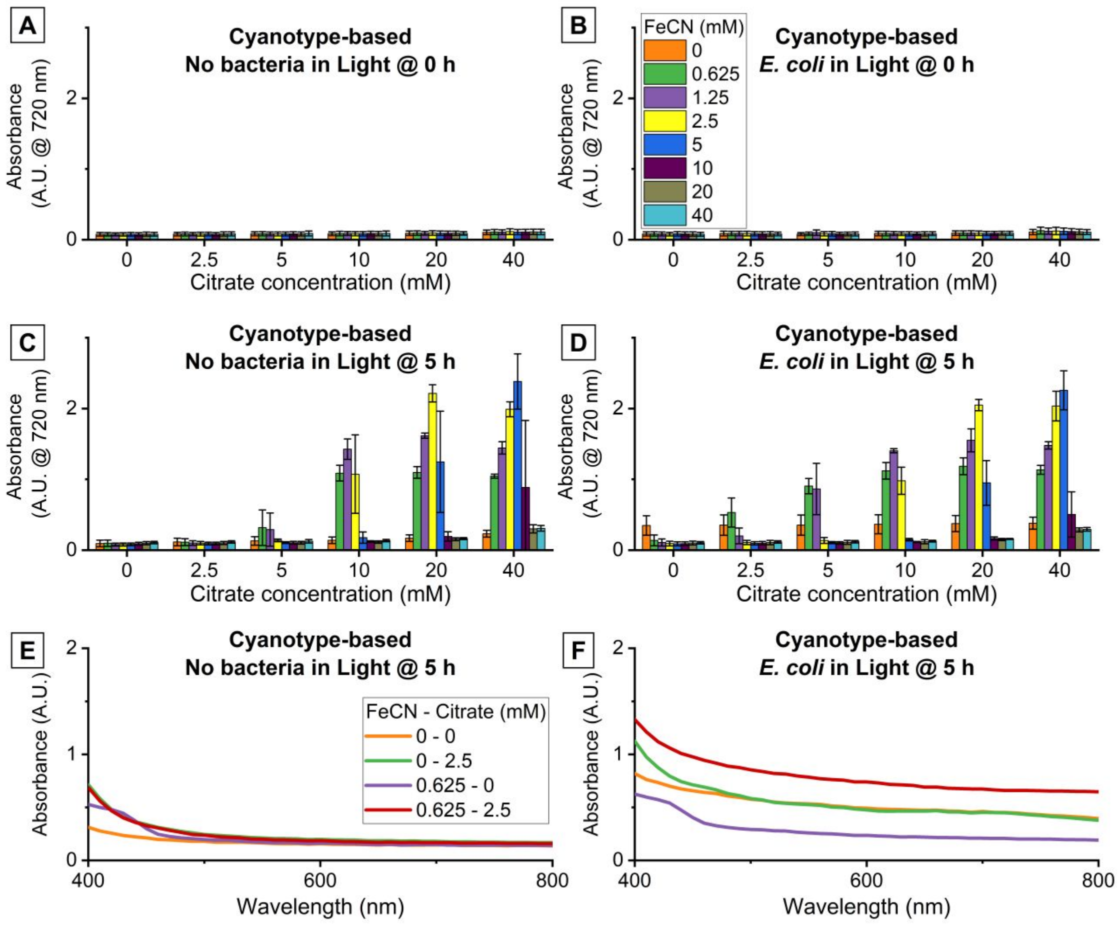

Fig. S5: Cyanotype optimization under light conditions. *A) No bacteria are present, with an incubation time of 0 hours. B) 5x10^5 CFU/mL of E. coli ATCC25922 with an incubation time of 0 hours. C) No bacteria are present, with an incubation time of 5 hours. D) 5x10^5 CFU/mL of E. coli ATCC25922 with an incubation time of 0 hours. E) Absorbance spectra for 4 conditions, without bacteria, after an incubation time of 5 hours. F) Absorbance spectra for 4 conditions, with 5x10^5 CFU/mL of* E. coli *ATCC 25922, after an incubation time of 5 hours. Each group of columns refers to a single ferric ammonium citrate concentration. Each colour corresponds to a specific ferricyanide concentration. Data is plotted at a wavelength of 720 nm for A – D. (n=3)*

When the sample was continuously illuminated (5 hours of constant irradiation with visible light), the spectra changed dramatically. In the absence of bacteria, PB formation was only observed for precursor solutions with citrate concentrations above 10 mM and when combined with specific FeCN concentrations. Concretely:

0.625 – 2.5 mM FeCN for 10 mM citrate;

0.625 – 5 mM FeCN for 20 mM citrate;

0.625 – 10 mM FeCN for 40 mM citrate.

Furthermore, maximum absorbance occurs at a different concentration for each citrate concentration,

1.25 mM FeCN for 10 mM citrate;

2.5 mM FeCN for 20 mM citrate;

5 mM FeCN for 40 mM citrate.

It was thus evident that a ratio of 8-1 (citrate:FeCN) was optimal for PB formation, which differed from conventional cyanotype processes for photochemical production of PB where a 3 – 1 ratio (approximately 760 mM citrate and 240 mM FeCN) is reported as optimal. Also relevant, a minimum of 10 mM citrate was necessary to produce detectable PB concentrations within 7 hours of experiment.

Three conditions were identified where the presence of bacteria had a significant increment on PB formation when compared to controls without bacteria, namely:

2.5-0.625 mM (citrate-FeCN);

5-0.625 mM (citrate-FeCN);

5-1.25 mM (citrate-FeCN).

All of them presented low cyanotype precursor concentrations and a molar ratio between 4 and 8. From the three, the first one was discarded since the increase was not significantly higher than the biomass itself.

<u>Supplementary information S4:</u> Evaluation of the cross-reactivity of the cyanotype-based reagent with serum and blood components.

This section shows a first step towards testing in real samples, using human serum (obtained from Sigma Aldrich) and pig's blood. The experiments performed in serum (**Fig. S6**) and blood (**Fig. S7**) were conducted under the experimental conditions optimized before (2.5 mM citrate – 0.625 mM FeCN) as a proof-of-concept. Serum and blood were spiked with bacterial samples and diluted half with the precursor solution (in MH). Since the pH of the solution was crucial for the assay (PB dissolves at basic pH), three separate experiments were performed where the pH of the MH was adjusted to 6.6, 6.1 and 5.5 respectively.

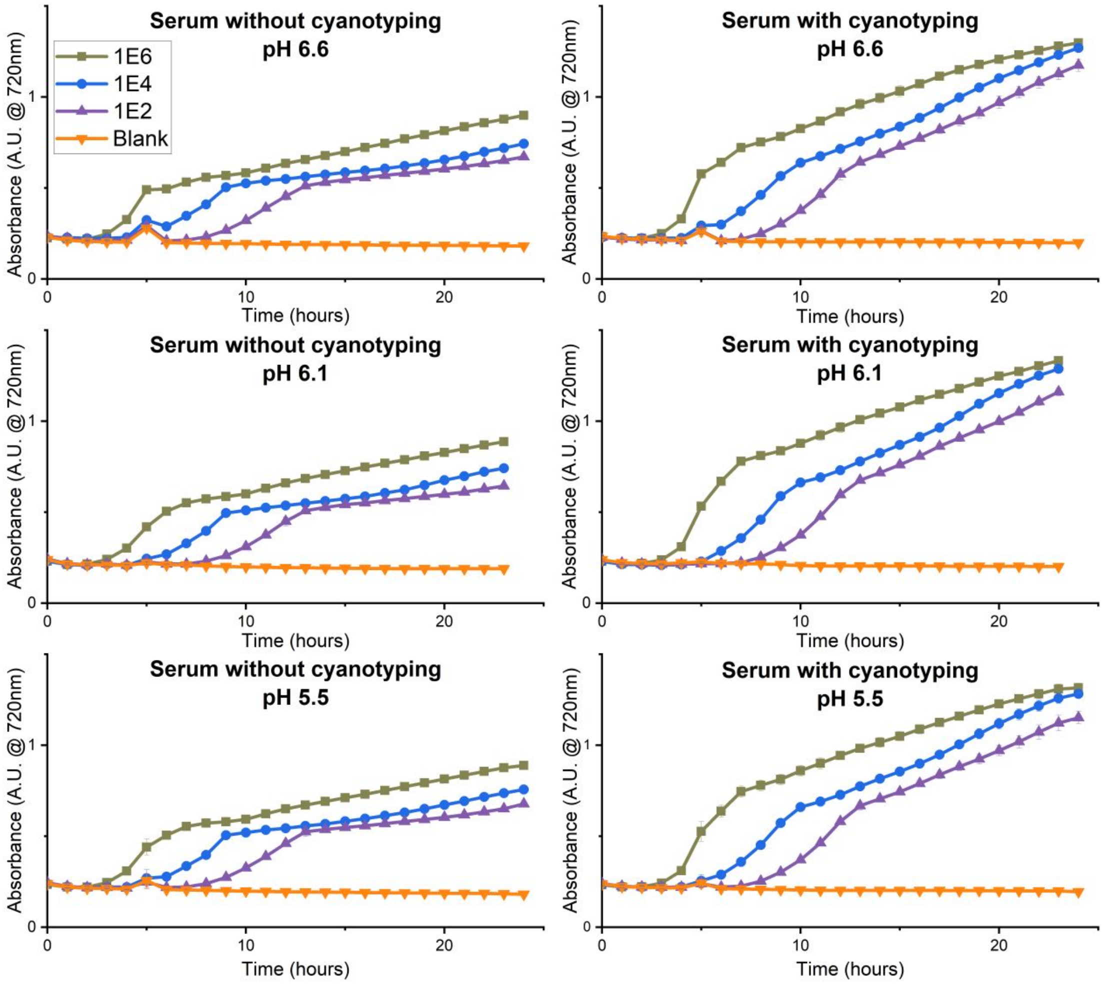

Fig. S6. Sensitivity of the cyanotyping assay performed in human serum. *E. coli ATCC 25922 was used as model organism. The measurement was performed using a 1:1 serum - MH dilution, under continuous illumination for up to 5 hours. For the cyanotyping, a 2.5 mM iron (III) citrate and a 0.625 mM ferricyanide were used. Results were measured and plotted at 720 nm. (n=6).*

Even though three very distinct samples of MH were prepared, the resulting pH after mixing with the serum was 7.0 ± 0.1 due to their buffering effect resulting in no significant differences between experiments. Results from the experiment are plotted in **Fig. S6.** Both experiments (i.e. with and without PB precursors) showed a conventional bacterial proliferation curve with the lag, exponential and stationary phases clearly visible. The main difference between samples was again the absorbance magnitude, this being higher in the samples containing precursor reagents due to the signal amplification associated to PB formation. The absence of the sudden jump after 3 – 4 hours of incubation in the samples containing PB precursors may be associated to some matrices effects. Serum components may capture some of the components of the assay and therefore, the proportion and concentration of precursors should be optimized in this complex matrix. Anyway, samples did not show cross-reactivity and thus the assay may be conducted directly in serum samples without any pretreatment.

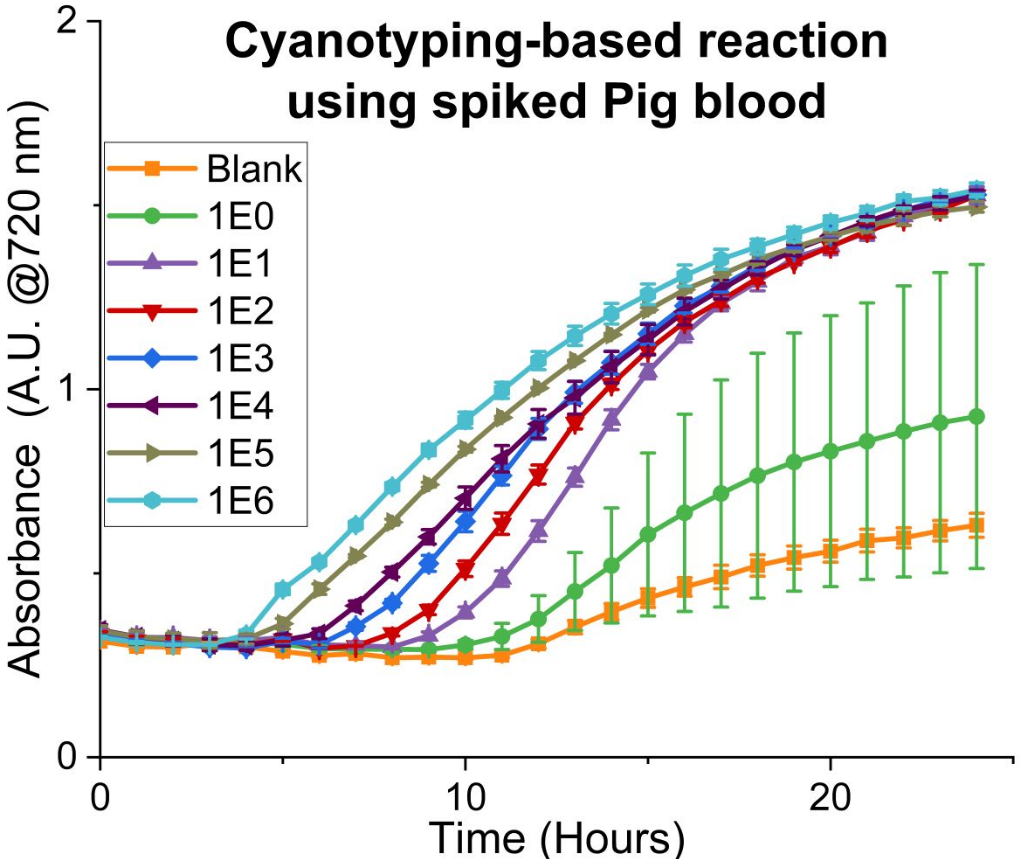

Fig. S7. Sensitivity of the cyanotyping assay performed in pig's blood. *E. coli ATCC 25922 was used as model organism. The measurement was performed using a 1:1 blood - MH dilution, under continuous illumination for up to 5 hours. For the cyanotyping, a 2.5 mM iron (III)*

citrate and a 0.625 mM ferricyanide were used. Results were measured and plotted at 720 nm. (n=3).

Finally, a cyanotype based test was also performed in complete blood samples (**Fig. S7**). As with serum, all bacterial concentrations followed a conventional proliferation curve with the amplification associated to PB formation. It confirmed that the assay could be performed in complete blood without interference of any of the blood components, opening the possibility for direct bacterial detection in blood samples. One of the aspects to be improved was reagents proportion since, as also observed in the case of serum, the initial sudden increase that reported on the presence of low bacterial concentration, was not obtained in this case. Two more aspects should be remarked from these results. First, as commented in other situations with low bacterial concentrations, the 10^1 CFU/mL sample converted the measurements into a probability assay, resulting in a growth curve with big error bars. Second, the blank line was not completely stable but presented some increase that may be due to some late reaction (after 11h of reaction) between precursor components to produce PB. Therefore, the results did not indicate cross-reaction between precursor reagents and matrix components allowing bacterial detection in complex matrices such as serum and blood directly and without any pre-treatment.
